# Supplementary material for: Alice in Suicideland: Exploring the Suicidal Ideation Mechanism through the Sense of Connectedness and Help-Seeking Behaviors
Source: Int J Environ Res Public Health. 2021 Apr 1;18(7):3681. doi: 10.3390/ijerph18073681 (PMC8037954; doi:10.3390/ijerph18073681)
Supplement: Supplementary file 1 [file ijerph-18-03681-s001.pdf]

--------- Code for Alice in Suicideland ---------

Data preparation

```
data1<-read.csv("C:/Users/…/data.csv",header = TRUE)
keeps <- c("Suicide", "inter_dom", "TCC","FormalHelp","InformalHelp")
data1 <- data1[keeps]
data1<-na.omit(data1)

library(bayesvl)
library(ggplot2)
library(cowplot)
```

Model 1

```
model1<-bayesvl()
model1<-bvl_addNode(model1,"Suicide","binom")
model1<-bvl_addNode(model1,"inter_dom","binom")
model1<-bvl_addNode(model1,"TCC","norm")

model1<-bvl_addNode(model1,"TCC_inter_dom","trans")

model1<-bvl_addArc(model1,"inter_dom","TCC_inter_dom","*")
model1<-bvl_addArc(model1,"TCC","TCC_inter_dom","*")

model1<-bvl_addArc(model1,"TCC","Suicide","slope")
model1<-bvl_addArc(model1,"TCC_inter_dom","Suicide","slope")

bvl_bnPlot(model1)


# Generate Stan code

model_string1 <- bvl_model2Stan(model1)
cat(model_string1)


# Model Fit

model1<-bvl_modelFit(model1, data1, warmup = 2000, iter = 5000, chains = 4,cores = 4)


# Figure 2 visualization

model1_figure1<-bvl_plotTrace(model1)
model1_figure2<-
bvl_plotIntervals(model1,c("b_TCC_Suicide","b_TCC_inter_dom_Suicide"))+theme_bw()
plot_grid(model1_figure2, model1_figure1,nrow = 2, labels = c('A', 'B'))
```

Model 2

```
model2<-bayesvl()
model2<-bvl_addNode(model2,"InformalHelp","norm")
model2<-bvl_addNode(model2,"TCC","norm")
model2<-bvl_addNode(model2,"inter_dom","binom")

model2<-bvl_addNode(model2,"TCC_inter_dom","trans")

model2<-bvl_addArc(model2,"inter_dom","TCC_inter_dom","*")
model2<-bvl_addArc(model2,"TCC","TCC_inter_dom","*")

model2<-bvl_addArc(model2,"TCC","InformalHelp","slope")
model2<-bvl_addArc(model2,"TCC_inter_dom","InformalHelp","slope")

bvl_bnPlot(model2)


# Generate Stan code

model_string2 <- bvl_model2Stan(model2)
cat(model_string2)


# Model Fit

model2<-bvl_modelFit(model2, data1, warmup = 2000, iter = 5000, chains = 4,cores = 4)


# Figure 3 visualization

model2_figure1<-bvl_plotTrace(model2)
model2_figure2<-
bvl_plotIntervals(model2,c("b_TCC_InformalHelp","b_TCC_inter_dom_InformalHelp"))+theme_bw()
plot_grid(model2_figure2, model2_figure1,nrow = 2, labels = c('A', 'B'))
```

Model 3

```
model3<-bayesvl()
model3<-bvl_addNode(model3,"FormalHelp","norm")
model3<-bvl_addNode(model3,"TCC","norm")
model3<-bvl_addNode(model3,"inter_dom","binom")

model3<-bvl_addNode(model3,"TCC_inter_dom","trans")

model3<-bvl_addArc(model3,"inter_dom","TCC_inter_dom","*")
model3<-bvl_addArc(model3,"TCC","TCC_inter_dom","*")
```

```
model3<-bvl_addArc(model3,"TCC","FormalHelp","slope")
model3<-bvl_addArc(model3,"TCC_inter_dom","FormalHelp","slope")

bvl_bnPlot(model3)


# Generate Stan code

model_string3 <- bvl_model2Stan(model3)
cat(model_string2)


# Model Fit

model3<-bvl_modelFit(model3, data1, warmup = 2000, iter = 5000, chains = 4,cores = 4)


# Figure 4 visualization

model3_figure1<-bvl_plotTrace(model3)
model3_figure2<-
bvl_plotIntervals(model3,c("b_TCC_FormalHelp","b_TCC_inter_dom_FormalHelp"))+theme_bw()
plot_grid(model3_figure2, model3_figure1,nrow = 2, labels = c('A', 'B'))
```

Model 4

```
# Model construction

model4<-bayesvl()
model4<-bvl_addNode(model4,"Suicide","binom")
model4<-bvl_addNode(model4,"FormalHelp","norm")
model4<-bvl_addNode(model4,"InformalHelp","norm")

model4<-bvl_addArc(model4,"FormalHelp","Suicide","slope")
model4<-bvl_addArc(model4,"InformalHelp","Suicide","slope")

bvl_bnPlot(model4)


# Generate Stan code

model_string4 <- bvl_model2Stan(model4)
cat(model_string4)


# Model Fit
```

```
Model4<-bvl_modelFit(model4, data1, warmup = 2000, iter = 5000, chains = 4,cores = 4)
```

Figure 5 visualization

```
model4_figure1<-bvl_plotTrace(model4)
model4_figure2<-
bvl_plotIntervals(model4,c("b_FormalHelp_Suicide","b_InformalHelp_Suicide"))+theme_bw()
plot_grid(model4_figure2, model4_figure1,nrow = 2, labels = c('A', 'B'))
```
